# A Review on the Quality of Colonoscopy Reporting

**DOI:** 10.1155/2016/9423142

**Published:** 2016-04-26

**Authors:** Robyn S. Sharma, Peter G. Rossos

**Affiliations:** ^1^Department of Internal Medicine, University of Toronto, Toronto, ON, Canada; ^2^Division of Gastroenterology, University of Toronto, Toronto, ON, Canada

## Abstract

Colonoscopy reports are important communication tools for providers and patients with potential to serve as information sources for research, quality, performance, and resource management. Despite decades of work, studies continue to indicate that colonoscopy reports are often incomplete. Although electronic medical records (EMRs) and databases can address this problem, costs, workflow, and interoperability (difficulty exchanging information between systems) continue to limit adoption and implementation of endoscopy EMRs in Canada and elsewhere. Quality and reporting guidelines alone have proven to be insufficient. In this review we have derived and applied five key themes to challenges in the current state of colonoscopy reporting and propose strategies to address them.

## 1. Introduction

Colonoscopy is integral in the detection and prevention of colorectal cancer (CRC), the second leading cause of cancer death in North America. The American Society for Gastrointestinal Endoscopy (ASGE) initially published colonoscopy quality guidelines in 1988 and most recently updated them in January 2015 [1, 2]. The Canadian Association of Gastroenterology published national consensus guidelines on safety and quality indicators in endoscopy in 2012 [3]. Colonoscopy performance is operator-dependent with wide variability in quality and cancer screening effectiveness between endoscopists [4]. Analysis of Canadian administrative data has associated quality measures and endoscopist specialty with missed CRC after colonoscopy [5, 6]. Standardized colonoscopy reporting and data management are important requirements for quality improvement [3, 7]. In the United States, the Quality Assurance Task Group of the National Colorectal Cancer Roundtable incorporated ASGE guidelines into their colonoscopy reporting and data system (CO-RADS) [8]. Despite these efforts adoption of standardized EMRs and databases remains poor in Canada and other jurisdictions. This limits opportunities to advance quality of care programs and optimize operational efficiencies. The following review identifies five key themes currently related to colonoscopy reporting practices along with strategies to address these challenges.

CO-RADS 25 key data quality indicators for colonoscopy reports are as follows.

*Patient Demographics and History*

- age,
- sex,
- other: anticoagulation, antibiotic prophylaxis required, implantable defibrillator, or pacemaker present.

*Assessment of Patient Risk and Comorbidity*

- ASA classification.

*Procedure Indication(s)*

- date of last colonoscopy,
- previous most advanced histological lesion,
- family history of CRC, adenoma, or inherited syndrome,
- reason for examination.

*Procedure: Technical Description*

- date and time,
- sedation with medication names and dosages,
- extent of examination,
- duration,
- documentation of cecal landmarks,
- retroflexion,
- bowel preparation (type and quality).

*Colonoscopic Findings*

- mass/polyp (location, size, morphology, and method of removal or biopsy),
- other abnormalities.

*Assessment*

- based on history and colonoscopy findings.

*Interventions/Unplanned Events*

- type of event ± intervention.

*Follow-Up Plan*

- immediate follow-up and discharge plan (further tests, referrals, changes in medications, and follow-up appointments),
- recommendation for follow-up colonoscopy and tests.

*Pathology.*

## 2. Methods

PubMed and Embase literature searches were performed for English language articles, published after 1970 using the terms “colonoscopy”, “quality”, and “reporting”. Initial searches identified approximately 250 articles, all of which were further screened for relevance. We also examined cited references and investigated those that were pertinent. In addition, we reviewed the Canadian Association of Gastroenterology and the American Gastroenterology Association guidelines on colonoscopy and colorectal cancer screening. Thirty articles were ultimately selected; when possible we chose the most recently published, as well as those with the highest level of evidence.

After reading the references from our literature search, the authors met and jointly identified five consensus colonoscopy reporting themes related to quality advancement in colonoscopy. These were based on gaps in quality that were recognized after surveying the literature.

These themes were further developed by performing focused literature searches for each topic.

## 3. Discussion

The five identified themes are as follows:

1. The need for standardized data models and templates.
2. The need for endoscopists to understand the value of complete and accurate documentation for effective clinical communication.
3. The need for standardized terminology.
4. The need for endoscopist performance feedback.
5. The need for appropriate health system use of data.

The themes are each discussed along with proposed strategies to address them.

### 3.1. The Need for Standardized Data Models and Templates

Studies have repeatedly demonstrated that colonoscopy reports are often incomplete [9–11]. Despite well-established documentation guidelines, there is considerable variability in colonoscopy report quality between individuals, endoscopy units, and geographic regions [12–14]. A study of reports from more than one hundred academic endoscopy centres throughout the United States revealed that key elements such as preparation quality and diagnostic interpretation were missing in 40% and 58% of cases, respectively [10]. An examination of community fee-based colonoscopy reports submitted to the US Veterans Administration revealed low overall completeness attributed to a lack of knowledge of reporting guidelines and/or poor agreement regarding reporting elements. It was suggested that automated endoscopy software may improve reporting compliance but may not completely standardize reporting quality [12].

Singh et al. [13] recently audited a large volume of dictated colonoscopy reports from community and academic centres in Manitoba and found that many were deficient in reporting key quality indicators. Only 20% documented bowel preparation quality and less than 10% specified the preparation used. When reporting polyps, only 34% described morphology, 10% omitted intervention details, and 2% neglected location [13]. Numerous other audits have demonstrated similar rates of report deficiency [9, 11, 14–18]. It is clear that published best practice colonoscopy reporting guidelines remain poorly adopted in many jurisdictions.

Improved report completeness has been demonstrated in areas with established electronic reporting systems [8, 19, 20]. In Canada, the Montreal General Hospital [15] and Alberta Health Services [21] have reported successful quality monitoring and improvement initiatives based on this approach.

*Recommendation.* Standardized data reporting models and templates are required to integrate best practices and data collection at the point of care.

### 3.2. The Need for Endoscopists to Understand the Value of Complete and Accurate Documentation for Effective Clinical Communication

Colonoscopy reports are essential communication tools for multiple stakeholders. Referring physicians require summarized findings, diagnoses, interventions, therapeutic, and follow-up recommendations. Subsequent endoscopists expect documented procedural details (i.e., sedation, instruments used, etc.), as well as any special considerations or difficulties that may have been encountered [22]. Effective “handover” is especially relevant for colorectal cancer screening programs where patients receive care from different endoscopists over time.

Literature review suggests that procedure documentation has been improving at a very slow rate and remains suboptimal even in leading organizations. In 1991, Mai et al. [23] reviewed colonoscopy reports and discovered that even after the implementation of ASGE reporting guidelines only 28.7% included a follow-up plan. In 2002 Robertson et al. [10] found that 59% of reports in research-affiliated facilities included a procedure interpretation and plan. More recently at the Mayo Clinic, follow-up recommendations and screening intervals were found in only 81% of colonoscopy reports [24]. In addition to incomplete summaries and recommendations the procedural details provided are often insufficient to determine appropriate surveillance intervals [8, 12, 15, 17]. This presents challenges for primary and referring physicians and is perceived as a significant barrier to effective care [25]. Key procedural details are especially relevant when suggested surveillance intervals exceed published guidelines; recommendations should be included in the report along with the rationale for deviating from protocol [26].

*Recommendation.* Professional organizations, referring physicians, training programs, and payers must continue to emphasize the need for and establish policies related to the timely and accurate reporting of colonoscopy procedures. Concise and complete colonoscopy reports prevent unnecessary procedures and facilitate appropriate follow-up intervals.

### 3.3. The Need for Standardized Terminology

The need for standardized terminology has been recognized by endoscopists for decades. In 1994, the European Society for Gastrointestinal Endoscopy (ESGE) developed the Minimal Standard Terminology (MST) with the goal of establishing a common vocabulary and structure for computerized endoscopy reporting systems [27, 28]. The MST details a selection of terms and descriptors for procedural indications, findings, anatomy, endoscopic diagnosis, and adverse events. Despite this excellent foundation, the goal remains largely unrealized in general practice. Li et al. [9] sampled colonoscopy reports throughout the state of Maryland and found considerable variation among endoscopists in their use of terminology to describe similar lesions. For example, 10 mm polyps were reported as “small” by some endoscopists and “large” by others and descriptors related to the bowel preparation varied, making comparison and interpretation difficult.

Although the Boston Bowel Preparation Scale (BBPS) is the most commonly used and validated scoring system in research settings [29], it is rarely incorporated in clinical endoscopic reports [30]. Educational programs involving brief online tutorials have successfully increased adoption of the BBPS in both US [31] and China [32]. Other validated grading systems include the Aronchick, Ottawa, and Chicago Bowel Preparation Scales [33–35]. The wide variability in bowel preparation reporting has significant impact on accuracy, safety, and system costs [11, 13, 17].

*Recommendation.* Further work is required to achieve and implement standard terminologies and classifications for the purposes of clarity, data analysis, and clinical decision-making. This process requires clinical leadership with ongoing support and commitment.

### 3.4. The Need for Endoscopist Feedback

Endoscopists require data to understand and continuously improve the quality of their personal reporting and procedure performance. The previously cited study by Mai et al. [23] from 1991 demonstrated that peer review through monthly quality assurance meetings reduced report deficiency rates from 91.6% to 33%, along with significant reduction in inappropriate indications for endoscopy. A more recent study was also able to achieve positive results through a 10-minute documentation completeness compliance rate review incorporated into their monthly staff meetings [24].

Canadian analysis of administrative data associated endoscopist quality measures with postcolonoscopy colorectal cancer [5]. Quarterly report cards and email messaging informing endoscopists of their individual performance data are simple interventions correlated with significant improvement in quality indicators, such as cecal intubation and adenoma detection rates [36–38]. Periodic point of care audits have been shown to supply a basis for evaluating variation while providing opportunities to improve clinical practice [36, 38]. These audits allow for a more realistic and clinically relevant appraisal compared to retrospective and planned interval reviews [11, 39].

In some centres, frequent audits of daily clinical practice reports have become a standard of care [40]. The Canadian Association of Gastroenterology led by Armstrong et al. [41] developed the Practice Audit in Gastroenterology (PAGE) program, enabling participants to evaluate their performance and compare it to pooled peer data. The program was well received as a tool for physicians to gather feedback regarding their personal practice and performance [42]. Evidence also supports the use of indicator reporting by trainees. A simple web-based tutorial significantly improved knowledge of quality performance measures among gastroenterology trainees, who at baseline had a very low rate of understanding [43].

*Recommendation*. Standardized reporting of performance indicators provides important quality improvement feedback for both trainees and experienced endoscopists. Systems and processes are required to provide this data in a timely and accurate manner.

### 3.5. The Need for Appropriate Health System Use of Data

Payment for performance is increasingly considered and applied to address rising health system utilization and costs [44]. To date most colonoscopy reporting practices do not support robust quality control initiatives [45], despite published guidelines on measurable outcomes and evaluation standards [46, 47]. System level monitoring of point of care data is required to identify and address both overutilization in low risk patients and appropriate access to care in those with high risk endoscopy findings and indications [26, 48]. There is also a great deal of disparities regarding utilization of colorectal cancer screening, which have been shown to be correlated to increased mortality [49, 50]. Large-scale analyses are required in order to identify and enact strategies to address such inequalities.

Quality reporting in colonoscopy is increasingly required to maintain public confidence. Health system management depends on metrics that are continually evaluated and associated with clinical outcomes and benchmarks [51]. Failure to allocate scarce resources based on access requirements and care quality can lead to misaligned incentives and poor outcomes.

*Recommendation*. Payment reform and public reporting depend on accurate data and relevant metrics. Standardized reporting and ongoing commitment to outcomes research and quality improvement are required to optimally allocate resources and support public trust.

## 4. Conclusion

We have identified a number of persistent deficiencies in current colonoscopy reporting. Quality, safety, and continuous improvement require a team approach with performance monitoring among primary care providers, endoscopists, group practices, and health systems [52]. Accurate and timely data collection, greater transparency, and analytics depend on point of care systems that are usable, efficient, and interoperable [53]. Standardized reporting models and templates can improve report completeness, provide timely communication, prevent unnecessary procedures, and enable appropriate follow-up [20].

Adoption barriers to endoscopy EMRs include costs, workflow considerations, lack of optimized content, and inability to incorporate evolving clinical best practice. Systematic approaches to design and clinical consensus can address these gaps to develop and support more effective and affordable endoscopic electronic reporting systems [54]. There is timely opportunity to expand national and international collaboration on standardized colonoscopy reporting, terminology, key quality indicators, and follow-up protocols. Structured, standardized electronic reporting systems and databases can more effectively serve as the thread to unite multiple areas of continuous quality improvement in colonoscopy.
